# Lexical selection in the semantically blocked cyclic naming task: the role of cognitive control and learning

**DOI:** 10.3389/fnhum.2014.00009

**Published:** 2014-01-27

**Authors:** Jason E. Crowther, Randi C. Martin

**Affiliations:** Department of Psychology, Rice UniversityHouston, TX, USA

**Keywords:** lexical selection, semantic blocking, aging, inhibition, individual differences

## Abstract

Studies of semantic interference in language production have provided evidence for a role of cognitive control mechanisms in regulating the activation of semantic competitors during naming. The present study investigated the relationship between individual differences in cognitive control abilities, for both younger and older adults, and the degree of semantic interference in a blocked cyclic naming task. We predicted that individuals with lower working memory capacity (as measured by word span), lesser ability to inhibit distracting responses (as measured by Stroop interference), and a lesser ability to resolve proactive interference (as measured by a recent negatives task) would show a greater increase in semantic interference in naming, with effects being larger for older adults. Instead, measures of cognitive control were found to relate to specific indices of semantic interference in the naming task, rather than overall degree of semantic interference, and few interactions with age were found, with younger and older adults performing similarly. The increase in naming latencies across naming trials within a cycle was negatively correlated with word span for both related and unrelated conditions, suggesting a strategy of narrowing response alternatives based upon memory for the set of item names. Evidence for a role of inhibition in response selection was obtained, as Stroop interference correlated positively with the change in naming latencies across cycles for the related, but not unrelated, condition. In contrast, recent negatives interference correlated negatively with the change in naming latencies across unrelated cycles, suggesting that individual differences in this tap the degree of strengthening of links in a lexical network based upon prior exposure. Results are discussed in terms of current models of lexical selection and consequences for word retrieval in more naturalistic production.

## Introduction

The ability of speakers to quickly and precisely select words from the mental lexicon is fundamental for the production of fluent and accurate speech. As such, the representational levels and processes involved when individuals produce single words has been a major focus in psycholinguistic research on language production. In the current study, we investigated the processes involved when speakers produce the name of a picture in the context of naming other semantically related or unrelated items. A number of lines of evidence indicate that when speakers attempt to produce the name of a picture, the names of other semantically related items become activated as well, thus requiring some mechanism to select the correct name from among these competitors. Additionally, some studies have reported evidence suggesting that cognitive control mechanisms are involved in the selection process, with inhibition often argued to play an important role (de Zubicaray et al., 2001, 2006; Guo et al., 2011; Shao et al., 2012).

**Figure 1:**
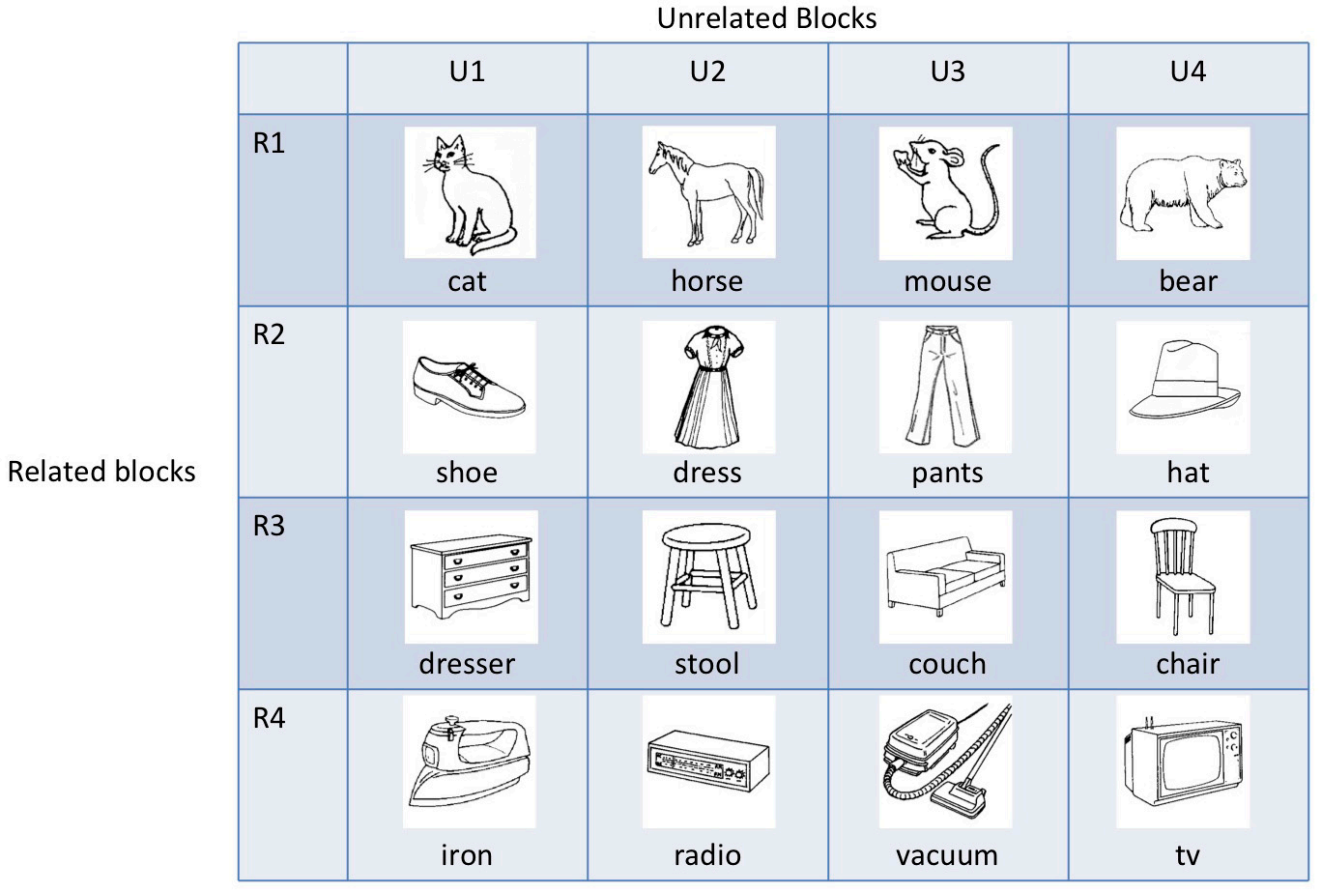
**Example of semantic blocked cyclic naming design with four items per category**.

We assessed the role of cognitive control mechanisms in picture naming using the semantically blocked cyclic naming task. In this task, subjects repeatedly name pictures in sets of either semantically related or unrelated pictures. In the typical administration of the task, subjects are presented with items in related and unrelated blocks, cycling several times through the items in each block, and each subject sees each item in both a related and unrelated context (e.g., Belke et al., 2005; Schnur et al., 2006). An example of a design with four items from each of four categories is presented in Figure 1, analogous to what was used in the Belke et al. study^1^. The items in each row would serve as the items in a related block and the items in each column would serve as the items in an unrelated block. For each cycle within a block, the same four items are presented in a different random order with some constraints on immediate repetition across cycles. In this fashion, all of the same items are named in both related and unrelated blocks, thus eliminating item-specific effects across conditions. Naming latencies are longer for the pictures from the semantically related than unrelated sets, with the difference either increasing across cycles of naming the picture sets or remaining constant. The largest increase in the semantic blocking effect occurs between cycle 1, where no difference or even a semantic facilitation effect is observed, to cycle 2, where semantic interference is observed. After cycle 2, the semantic interference effect increases more slowly (e.g., Schnur et al., 2006; Biegler et al., 2008) or stays about the same (e.g., Belke et al., 2005; Damian and Als, 2005; Navarrete et al., 2012). Some researchers have attributed the interference effect (often referred to as the “blocking effect”) to increasing activation of all of the competitors during the naming of the semantically related sets (Belke et al., 2005; Schnur et al., 2006). Due to the increase in the activation of competitors, some mechanism may be needed to select the target name, either by increasing the activation of the appropriate name or inhibiting the activation of competitors (Roelofs, 2003).

A contrasting account of semantic interference in the blocked cyclic naming task, one in which cognitive control mechanisms play little role, has been put forward by Oppenheim et al. (2010). According to their account, semantic interference results from an incremental learning process in which the selection of a word for productions results in long-lasting changes in the strengths of connections in a semantic-lexical network. Support for a learning-based account of semantic blocking effects comes from findings that semantic interference persists across the naming of semantically unrelated items or performing completely different experimental tasks (Damian and Als, 2005). Further, in a continuous naming paradigm, in which subjects name pictures from different categories presented in a pseudorandom order without blocking or repetition of items, Howard et al. (2006) found longer naming latencies for subsequent words from the same semantic category, with an approximately linear increase in latencies with each additional sampling from the same category. Importantly, these authors found that the increase in naming latencies was unaffected by the number of intervening words from different categories. The results reported by Damian and Als and Howard et al. are hard to accommodate by an assumption of temporary activation of semantically related names, instead favoring a mechanism with a more long-lasting effect.

Howard et al. (2006) presented computational modeling evidence which they argued demonstrated that any model of speech production which could accommodate the cumulative interference in the continuous naming paradigm had to have three properties: (1) shared activation, such that activation of the semantic representation of one word leads to activation of words that are semantically related, (2) competition, such that activation of a competitor words delays selection of the target word perhaps through lateral inhibition or a selection ratio, and (3) priming, such that previously produced representations persist, perhaps through the strengthening of semantic-lexical connections.

Oppenheim et al. (2010) built on the modeling effort of Howard et al. (2006) in the development of their incremental learning model. Their model incorporated the notion of shared activation, but differed principally in that learning not only led to priming of the selected word due to the strengthening of the semantic-lexical connections for selected words but also led to interference for the non-selected word due to the weakening of lexical-semantic connections for unselected but related words. For example, correct selection of the word “cat” for a picture of a cat would lead to a strengthening of the links between the semantic features of cat (e.g., has fur, four-legged) and the word “cat,” but would also lead to a corresponding weakening of links between those same semantic features and other semantically related words which were not selected (e.g., “horse,” “dog”).

**Figure 2:**
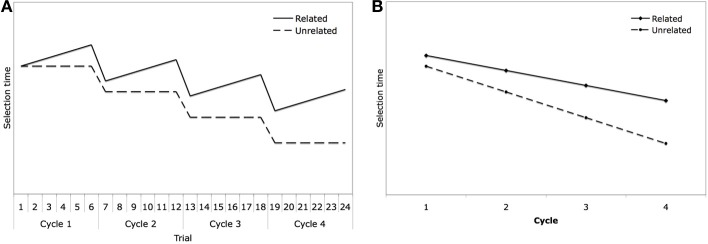
**Figures based upon Oppenheim et al. (2010) showing model performance (A) across trials within a block, and (B) across cycles within a block**.

Figures 2A,B (based on Figures 5a and 5b from the Oppenheim et al., 2010 paper) depict how their learning model accounts for the semantic relatedness effect and its growth across cycles. Figure 2A assumes that there are four cycles with six trials in each cycle within a related or unrelated block. Selection time (which would determine naming latency) is shown to grow across trials within a cycle for the related trials, but to stay flat for the unrelated trials. For related trials, the decrease in semantic-lexical connection weights for words semantically related to a selected word will mean that subsequent words in the cycle will be more difficult to name, and this difficulty will increase as more words from a semantically related set are named. For unrelated trials, the change in connection weights for the target word and words semantically related to it would not affect selection of the next word, as it is unrelated to the preceding word or other words in the same cycle. While the decrease in connection strengths between semantic features and unselected lexical representations leads to interference, the increase in connection strengths between semantic features and selected lexical representations leads to repetition priming when the same object is presented again for naming. In the learning model depicted in Figure 2A, repetition priming (i.e., the decrease in latencies between the last item of the preceding cycle and the first item in the next cycle) is similar for related and unrelated trials, though the effect for related trials is somewhat less due to the greater weakening of links for the target item due to naming of related items in the prior cycle. As a consequence of increasing latencies across cycles for the related items and the somewhat smaller repetition priming effect for the related trials, the size of the semantic relatedness effect grows across cycles, as shown in Figure 2B, where the data are collapsed across trials within each cycle. As is evident from the figure, the model predicts interference to be present in the first cycle, whereas no such effect, or even semantic facilitation, is often reported. Oppenheim et al. suggested that facilitation in the first cycle might be due to subject strategies, pointing to the fact that clear interference, rather than facilitation or lack of any effect, was observed in a study by Belke (2008) that used a secondary load, which may have interfered with such strategies.

Although the source of semantic interference is different in the incremental learning and activation approaches, it could still be the case that a control mechanism involving inhibition comes on line when several competing names are activated—whether the source of the competition is persisting activation or learning. For instance, the incremental learning model proposed by Oppenheim et al. (2010) assumes a booster mechanism which repeatedly increases the activation levels of all activated words until a winner can be selected, either through reaching a difference threshold between its activation level and that of other words or through an absolute threshold. One might assume individual differences in the efficiency of such a mechanism would modulate the size of semantic interference in naming, with greater efficiency resulting in a smaller increase in semantic interference.

In a recent review of the literature on semantic interference in naming, Belke (2013; Belke and Stielow, 2013) has proposed an account in which semantic interference may accumulate in an incremental manner due to bottom-up processes, along the lines of the incremental learning accounts, but in which top-down cognitive control plays a critical role in resolving semantic interference when subjects are able to use task information to bias selection of a particular target [see also Scott and Wilshire (2010) for a similar proposal]. Belke and Stielow noted that typically the size of the semantic interference effect does not increase beyond cycle 2, in contrast to the continual growth predicted by the incremental learning model (see Figure 2B). Further, Belke (2008) had shown that semantic interference in blocked cyclic naming increased with a concurrent digit load, suggesting a role for working memory in modulating semantic interference in this task, whereas Belke and Stielow reported no such effect in a continuous naming paradigm. While Belke and Stielow endorse a role for learning in contributing to semantic interference in naming, they argue that the inconsistencies between model predictions and actual data as well as inconsistencies in findings between naming paradigms can be explained by an appeal to top-down control which biases the activation of the items within the response set in the blocked cyclic task, and which would serve to offset the increasing competition derived from learning. They argue that carrying out this biasing requires subjects to maintain a representation of the task set and of the items in the current naming set, and thus interference resolution will be less efficient either for those with smaller working memory capacities or under conditions of working memory load. In line with this argument, they point to neuropsychological findings demonstrating a significant increase in latencies beyond the second cycle for patients with left inferior frontal damage (Schnur et al., 2006; Biegler et al., 2008) who have associated deficits in working memory (e.g., Biegler et al., 2008) or in selecting task-relevant responses (Thompson-Schill et al., 1998).

In the present study, we examined these various proposals by determining whether inhibitory abilities would in fact be related to the size of semantic interference effects in the blocked cyclic naming task. To the extent that selection from competitors is involved, one might predict that those with poorer inhibitory abilities would show larger interference effects. If selection is not a competitive process (e.g., Mahon et al., 2007; Oppenheim et al., 2010) then one would predict no relationship between semantic blocking interference and inhibitory abilities. Three measures of cognitive control abilities were employed. A standard word span task, in which subjects repeated back in order a list of words drawn from a closed set, was used to tap working memory capacity (Baddeley and Hitch, 1974). Since Belke (2008) demonstrated that a digit load increased the size of semantic interference in blocked cyclic naming, we expected that those with larger word spans would show less semantic interference. The other two measures assessed the abilities of individuals to select the correct representation from irrelevant, distracting representations. One task (Stroop; Stroop, 1935) was selected to tap response-distractor inhibition and the other (recent negatives; Monsell, 1978) the resolution of proactive interference. Prior findings have established that these are at least partially independent inhibitory abilities (Friedman and Miyake, 2004). Response-distractor inhibition involves the selection of one representation or response from competing representations or responses that are derived from distracting stimuli present in the environment. The standard Stroop task was used as our measure of this type of inhibition. Proactive interference, in contrast, derives from interference from persisting memory representations, and the recent negatives task (Monsell, 1978) was used as our measure. In this task, subjects are presented with a list of items followed by a probe, and must respond whether the probe was in the list of items or not. The critical manipulation is whether the probe was an item in the immediately preceding list, but not the current list, as compared to when it was not recently presented. Both younger and older adults were tested, as there is evidence that older adults have poorer inhibitory abilities and reduced memory span (Hasher and Zacks, 1988; Hasher et al., 2007). Thus, to the extent that these abilities play a role in word selection, age-related differences may be observed, with the older subjects showing larger effects. Further, including the older subjects should serve to increase the range of these cognitive control abilities, improving the ability to detect a relation between them and picture naming. Although Belke and Meyer (2007) failed to show an interaction between age and the size of the semantic blocking effect in single object naming, their sample size was fairly small, and the larger sample size used here may be sufficient to reveal such an interaction.

## Methods

### Participants

The subjects were 41 younger subjects (mean age: 25.6, range: 18–43 years old, 33 females, 8 males) who were students enrolled at the University of Houston-Clear Lake and 42 older subjects (mean age: 62.9, range: 45–80 years old, 32 females, 10 males) primarily from the Rice University Brain and Language Lab older subject database. Two of the older subjects were University of Houston-Clear Lake students who fell within the age range for older subjects. Students participated for course credit and older subjects were paid $20 in compensation. One older subject was later removed from the analyses because of outlying scores on a number of measures.

### Materials, design, and procedure

#### Word span

The administration of this task followed standard word span procedures (e.g., Baddeley and Hitch, 1974). The experimenter read aloud lists of words, drawn from a closed set of 10 words, at a rate of about one item per 1.5 s and the subject repeated back the list. List lengths began at one and increased to a maximum of 7 with 10 lists at each list length. Testing was discontinued when the subject recalled less than 50% of the lists correctly at a given length. Lists were counted incorrect if the subject failed to recall all the words, recalled them in the incorrect order, or produced an item not on the list. Word span was calculated as the list length at which recall was 50% based on linear interpolation between the lists lengths that spanned the 50% level. For example, if the subject scored 70% correct at list length five and 30% correct at list length six, the estimated span was 5 + ((70 − 50) / (70 − 30)) = 5.5. If recall never dropped below 50%, the subject was assigned a word span of 7.

#### Stroop

Subjects named the font color of a color word while ignoring its name. Subjects first completed 10 practice trials, followed by experimental trials consisting of 72 neutral trials consisting of a row of colored asterisks, 60 incongruent trials in which the ink color and color word differed, and 12 congruent trials in which the ink color and color word were the same. Trials were presented in the same random order for all subjects. The dependent measure was the difference between mean naming latencies in the incongruent and neutral trials.

#### Recent negatives

Subjects were presented auditorily with a list of three words followed by a probe word and indicated with a button press whether the probe word was in the list. There were 48 trials in which the probe word was in the list (positive trials). There were 48 negative trials divided equally into two types. On recent negative trials, the probe word had appeared in the immediately preceding list. On non-recent negatives, the probe word had not appeared in the current or immediately preceding lists. The proactive interference measure was the difference in reaction times for recent negative and non-recent negative trials. Each subject was presented with the same order of items.

#### Blocked cyclic naming

Stimuli were 128 line drawings, drawn from the International Picture Naming Norms (Szekely et al., 2004), with 8 exemplars from 16 semantic categories (see Appendix 2). Sixteen related and 16 unrelated sets of eight items each were created. Different list orders were created by a Roman square design so that no picture was repeated on successive trials. The task was divided into two halves, with each half containing eight related sets and eight unrelated sets, with related and unrelated blocks appearing in a different ABBA order (i.e., related, unrelated, unrelated, related or unrelated, related, related, unrelated order) for each subject. The items in related sets in one half were presented in unrelated sets in the other half, which was counterbalanced across subjects. Pictures were presented individually on a computer and naming latencies were recorded by a voice-key. In a practice session, subjects were presented with each picture along with a written name, which they read aloud. Experimental trials began with the presentation of a single picture. The subject would name the picture, which remain on the screen until the experimenter coded the trial as correct or not, after which a blank screen would be presented for 1 s before the onset of the next picture. The eight items within one related or unrelated set were presented in a blocked fashion with four cycles of presentation with each item presented once in each cycle. Thus, each block contained four cycles with each composed of eight trials, for a total of 32 trials in each block.

#### General procedure

Younger subjects completed the experiment in a single 2-h experimental session. For the younger subjects, the word span task was administered at the beginning of the experiment and the blocked cyclic naming task was administered last. The order of the other two tasks (Stroop and recent negatives) was counterbalanced across subjects. As the older subjects took longer to complete the tasks, the tasks were administered in two different sessions, with the first session consisting of the Stroop and blocked cyclic naming task, and the second session consisting of the recent negatives and span task. The sessions occurred about 1 week apart. All tasks, except the word span task, were administered on a computer using the PsyScope experimental software (Cohen et al., 1993).

#### Outlier analysis and data transformation

A two-step procedure was used to deal with outliers on all tasks with the exception of the span tasks [see Miyake et al. (2000) for a similar procedure]. First, upper and lower criteria were set for each task, with the lower bound being 300 ms for all tasks and the upper bound being 2000 ms for the Stroop tasks, 3500 ms for the recent negatives task, and 2500 ms for the Blocked Cyclic Naming task. Values more extreme than the criterion values were replaced with the criterion values. Second, the mean and standard deviations were calculated for each subject by task condition, and any response times more extreme than ± 3 standard deviations were replaced with the ± 3 standard deviation value^2^. These two procedures affected less than 2% of the data for both older and younger subjects across tasks, with the age groups differing by less than 1% for all tasks. After outliers were identified, all of the reaction time data were log-transformed. This was done in order to minimize any effects of general slowing for the older adults. That is, reaction time effects tend to be larger for subjects who are relatively slower (Salthouse and Hedden, 2002) and older subjects tend to be slower on average than younger subjects (Verhaeghen and Salthouse, 1997). Using log-transformed data for all of the effects would reduce the contribution of general slowing to any age-related effects.

#### Reliability analysis

In carrying out individual differences research, it is critical to determine the reliability of the measures going into correlations as the correlation between two measures is limited by their reliabilities (Conway et al., 2005). Specifically, the maximum possible correlation between two measures is the square root of the product of their reliabilities. Reliability reflects the degree to which a test gives consistent results, with values ranging from 0 (no reliability) to 1.0 (indicating perfect reliability). In the present study, a split-half reliability measure was computed, which reflects the internal consistency of the test, correlating the effect obtained from one half of the items with that of the other half. To compute this measure of reliability, the data for each subject were randomly divided into two halves, with equal numbers of data points in each condition in each half. A measure of performance was calculated for each subject from each of the two halves. Correlations between the two estimates for each measure were calculated, and then the Spearman-Brown Prophecy formula was applied to determine the reliability (Brown, 1910; Spearman, 1910).

## Results and discussion: blocked cyclic naming

Before addressing the individual differences effects, we first examined the pattern of responses in the blocked cyclic naming task to determine if the predicted interaction between age and semantic interference would be obtained. We also wished to examine specific aspects of the semantic interference effect related to the learning model of Oppenheim et al. (2010) and the top-down biasing proposal of Belke and Stielow (2013). Data from blocked cyclic naming are typically reported across cycle collapsing across both individual trials and blocks of the experiment (see Figure 2B). We also analyzed the results across trial with respect to the learning model (see Figure 2A). The model predicts increasing naming latencies across trials within a cycle and a repetition priming effect across cycles. According to the top-down biasing approach, the slope across trials should differ between cycle 1 and cycles 2–4, with the increase in interference across trials being greater between the first and second cycle than for later cycles. Finally, the data were analyzed across related and unrelated blocks of the experiment. This is particularly interesting for the unrelated blocks. As shown from the example in Figure 1, items within related blocks are semantically related, but they are not semantically related to items in different related blocks. In contrast, the items in unrelated blocks are not semantically related to one another within a block, but they are semantically related to items in different unrelated blocks. Thus, we would expect to see cumulative semantic interference across unrelated blocks but not across related blocks. If such findings are obtained, they would fit with findings from the continuous naming paradigm (e.g., Howard et al., 2006), and would further show that semantic interference persists across a very large number of trials.

### Effects across cycle and their interaction with age

Subjects made 2.34% (*SD* = 0.022) voice-key and 1.28% (*SD* = 0.012) naming errors. Given the small percentage of naming errors, no further analyses of errors were carried out.

**Figure 3:**
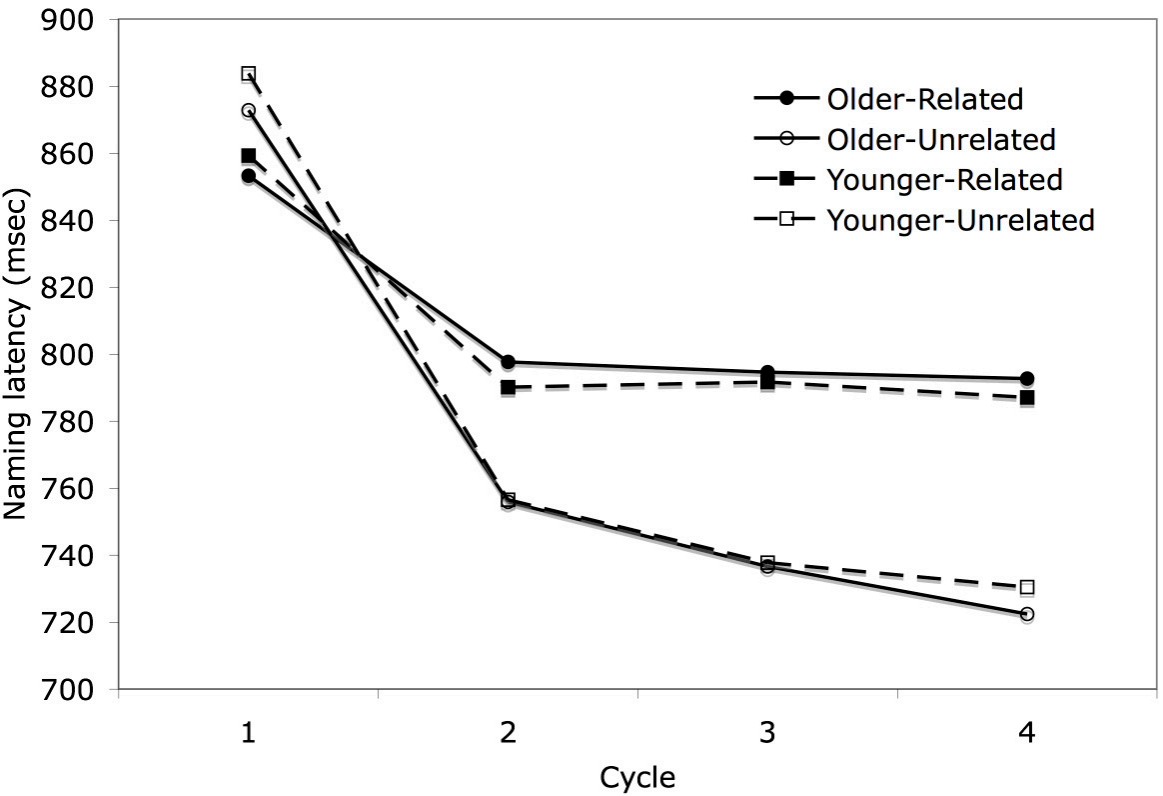
**Mean naming latency in the blocked cyclic naming task for both age groups by semantic blocking condition across cycles**.

As shown in Figure 3, the pattern for naming latencies replicated that from prior studies, with apparent facilitation in the first cycle followed by semantic interference in subsequent cycles. Latencies decreased over cycles for the unrelated trials, whereas latencies decreased from cycle 1 to 2 but then remained stable for the related trials. Subjects named items in the related condition more quickly than items in the unrelated condition on the first cycle [*t*_(81)_ = 5.72, *p* < 0.001], with the effect reversing for subsequent cycles [*t*_(81)_ = 19.53, *p* < 0.001, averaging across cycles 2–4]. The differences between naming latencies for the related and unrelated conditions were −22, 40, 56, and 68 ms across cycles 1–4, respectively. As is evident in the figure, the two age groups performed similarly, with mean naming latencies of 802 ms for the younger subjects and 792 ms for the older subjects. An initial overall analysis of naming latencies was carried out on the factors of age, relatedness, first half vs. second half, block, cycle, and trial. This analysis was only carried out with subjects as the random effect, but in later analyses collapsing across some of these factors, results are reported by both subjects and items. The overall ANOVA results are presented in Appendix 1. There was no main effect of age and only one interaction with age reached significance, which was an interaction between age, session half, and cycle. However, this interaction did not relate to any theoretical questions of interest and thus will not be discussed further.

**Figure 4:**
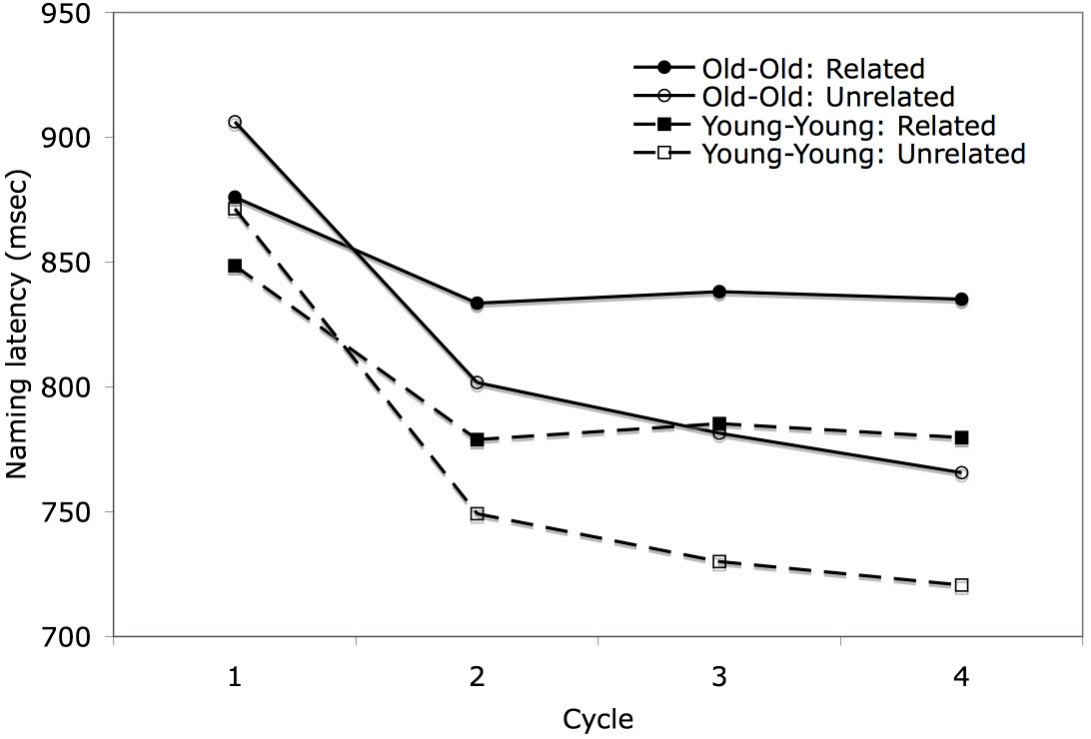
**Mean naming latency in the blocked cyclic naming task for subset of age groups by semantic blocking condition across cycles**.

Because of the relatively large range in ages within each age group, and the possibility that the older subjects as a whole were not old enough to reveal a different pattern between the groups, a separate analysis was performed on subsets of the two groups, including subjects younger than 25 years old (*N* = 28) in one subset and subjects older than 62 years (*N* = 20) in the other on the factors of relatedness and cycle. The results are shown in the Figure 4. The subset of older subjects had longer mean naming latencies (*M* = 830 ms) than the subset of younger subjects (*M* = 783 ms), but this difference was not significant [*F*_1(1,46)_ = 1.745, *p* = 0.193]. More importantly, no interactions with age group even approached significance [age group × relatedness: *F*_1(1,46)_ = 0.076, *p* = 0.784, age group × cycle: *F*_1(1,46)_ = 1.292, *p* = 0.280, age group × relatedness × cycle: *F*_1(1,46)_ = 1.118, *p* = 0.344]. Thus, the results replicated with a larger sample size the findings of Belke and Meyer (2007), who also failed to find a significantly greater semantic blocking effect for older than younger subjects in single object naming. Given the similarity of the findings for younger and older adults, age group was dropped as a factor in subsequent analyses.

### Slope effects across cycles and across trials within cycle

Having obtained the typical finding of an interaction of relatedness and cycle, we wished to determine if the slopes across cycles and across trials within cycles would conform to the patterns shown in Figures 2A,B as predicted by the learning model. As discussed earlier, the overall analyses documented semantic facilitation in cycle 1 and interference in cycles 2–4. Moreover, from Figures 3, 4, it is clear that there is a much steeper slope from cycle 1 to 2 than from cycle 2 onward for both the related and unrelated conditions. Thus, we calculated linear trends separately for cycles 1–2 and for cycles 2–4. For the related condition, naming latencies showed a significant 63 ms decrease between cycles 1–2 [*t*_1(81)_ = 12.67, *p* < 0.001] and then showed a non-significant decrease of 2 ms/cycle for cycles 2–4 [*t*_(81)_ = 1.26, *p* = 0.21]. For the unrelated condition, naming latencies showed a significant 122 ms decrease between cycles 1–2 [*t*_1(81)_ = 21.98, *p* < 0.001] and then showed a significant decrease of 14 ms/cycle for cycles 2–4, [*t*_(81)_ = 10.15, *p* < 0.001]. The greater decrease in response latencies between cycles 1 and 2 than from cycle 2 onward might be attributed in part to decreasing repetition priming over subsequent repetitions, a pattern which has long been observed in standard picture naming studies (e.g., Oldfield and Wingfield, 1965; Griffin and Bock, 1998). Oppenheim et al. (2010) noted that their model produced some decrement in repetition priming across subsequent repetitions, but this underestimated the large decrement reported in previous studies, and replicated here. The much greater decrease in latencies between the first two cycles than from cycle 2 onward is more readily accommodated by the top-down biasing approach (Belke and Stielow, 2013), as subjects would have encountered all the picture names in a set in the first cycle, which could then be used to narrow the response set in cycle 2. Of course, the lesser slope for the related than unrelated trials would be consistent with all approaches, as competition is assumed to be greater among semantically related items^3^.

**Figure 5:**
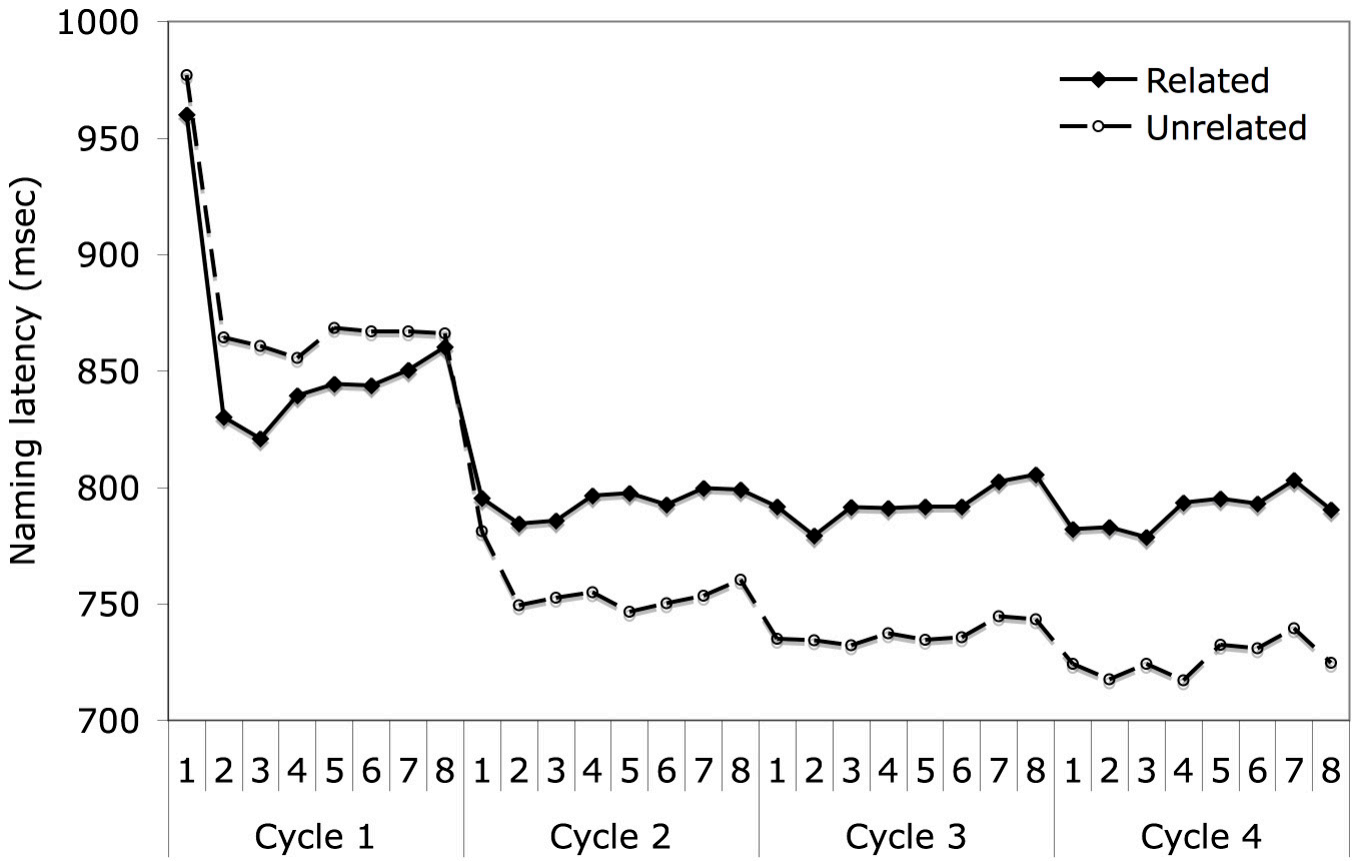
**Mean naming latency in the blocked cyclic naming task by semantic blocking condition across cycles and trials within a block**.

Next, we examined the change in the relatedness effect across trial within cycle to determine if the sawtooth function predicted by the learning model for the related trials (shown in Figure 2A) was obtained. The results are shown in Figure 5 and the results from overall ANOVAs by subjects and items with the factors of relatedness, trial, and cycle are shown in Table 1. As in the previous analysis, all of the main effects were significant, along with all of the two-way interactions between these variables.

**Table 1:**
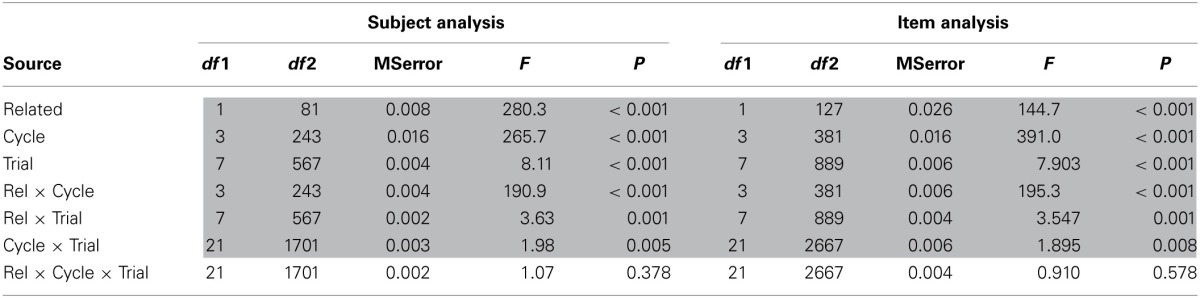
**ANOVA results for analysis of relatedness, cycle, and trial in the semantically blocked cyclic naming task**.

The shaded rows are those that were statistically significant at *p* ≤ 0.05.

Of more interest is an analysis that tested for linear trends across trials for the related and unrelated conditions. As is evident in Figure 5, cycle 1 differed from the other cycles in that, after the first trial, there was a semantic priming effect which diminished across trials, with the effect being about 30–40 ms at trials 2 and 3, but only 6 ms at trial 8 (related mean = 860 ms, unrelated mean = 866 ms). This facilitation on the first cycle would appear to conform to the results reported by Navarrete et al. (2012), which they attributed to short-term spreading activation at a semantic level which is eventually counteracted by increasing interference due to weakening of connections between lexical and semantic representations for items which are not initially named.

For both related and unrelated trials, naming latencies were noticeably longer on the first trial of the first cycle than on subsequent trials. Participants were given a short break between each block, and the longer naming latencies would presumably be attributed to a shifting effect when starting a new block. As this longer time would counteract any ability to see an increase in latencies across trials or cycles, the value for the first trial for the first cycle was replaced with the value for the second trial of the first cycle in this analysis. Overall, the linear trend across trials within cycle was significant [*F*_1(1,81)_ = 32.8, *p* < 0.001], with naming latencies increasing by 3.0 ms/trial. The relatedness × trial (linear) interaction was significant [*F*_1(1,81)_ = 19.8, *p* < 0.001], with the slope being significantly greater [*t*_1(81)_ = 3.86, *p* < 0.001] in the related (3.9 ms/trial) than the unrelated condition (2.2 ms/trial), with both of these slopes being significant. As evident in Figure 5, the linear trend was steeper in the first cycle than in the other three cycles and the statistical results confirmed this as the contrast of cycle 1 vs. cycles 2–4 × trial (linear) was significant [*F*_1(1,81)_ = 8.9, *p* = 0.004]. The three-way interaction of relatedness × cycle × trial (linear) was not significant [*F*_1(1,81)_ = 1.7, *p* = 0.20]. However, given that the top-down biasing approach predicts little or no increase in the interference effect after cycle 2, we thought it was important to examine the slope for the related compared to unrelated conditions in cycles 2–4 separately from cycle 1. The mean slopes in cycles 2–4 were 1.7 ms/trial for the related condition and 0.6 ms/trial for the unrelated condition, which were significantly different from each other [*t*_1(81)_ = 3.84, *p* < 0.001], substantiating that there was a small, but significant increase in the interference effect of 1.1 ms per trial (1.7 – 0.6 ms) even when the data from cycles 2–4 were examined alone.

Thus, the patterns of latencies were consistent with those predicted by the learning model in some respects, but differed in others. In contrast to predictions, there was a semantic priming effect within the first cycle, but latencies did increase more for the related than unrelated trials within the first cycle such that by the end of the first cycle very little semantic priming effect remained. The greater positive slope in the related trials than unrelated trials was predicted by the learning model, though the existence of a significant positive slope in the unrelated trials was not. Also, in the learning model, the slope for the related trials appears nearly equivalent within each cycle (as shown in Figure 2A); however, in our results, the slope across both the related and unrelated trials was greater in cycle 1 (ignoring the first trial) than in cycles 2–4. Thus, lesser slope of increase in latencies in both the related and unrelated trials after the first cycle would be consistent with the top-down biasing approach (Belke and Stielow, 2013) in which knowledge of the response sets after the first cycle counteracts interference in naming.

Finally, in the learning model, the bulk of the semantic interference effect derives from the steeper slope for the related than unrelated trials, although there is a lesser contribution from a smaller repetition priming effect for the related than unrelated trials. In our results, given that the latencies for the related and unrelated conditions were quite similar at the end of cycle 1, one can ask how much of the semantic interference effect can be attributed to the greater slope for the related trials. As the difference in slopes between the related and unrelated conditions for cycles 2–4 was 1.1 ms per trial (i.e., 1.7 ms/trial for related trials − 0.6 ms/trial for unrelated trials), the 24 trials after the end of cycle 1 would add 26 ms (i.e., 24 trials × 1.1 ms/trial) more for the related than unrelated trials. The difference in latencies between the related and unrelated conditions was -6 ms at the end of cycle 1 and thus the interference effect would be predicted to be 20 ms at the end of the 4th cycle based solely on the difference in slopes. However, this value is only about one-third of the actual value at the last trials of cycle 4 (66 ms). Thus, there is an additional effect that contributes substantially to the size of the interference effect. This additional effect can be seen in Figure 5 as arising mainly from a much smaller decrease in naming latencies for the related than unrelated trials specifically in going from cycle 1 to cycle 2—that is, the latency difference between trials 7 and 8 of cycle 1 and trials 1 and 2 of cycle 2 is about 36 ms larger for the unrelated than the related trials. For the other cycles, the decrease in latencies is similar for the related and unrelated trials. Thus, some factor is coming into play primarily in the transition between cycles 1 and 2 that plays a major role in the size of the semantic blocking effect.

### Effects across blocks and task halves

**Figure 6:**
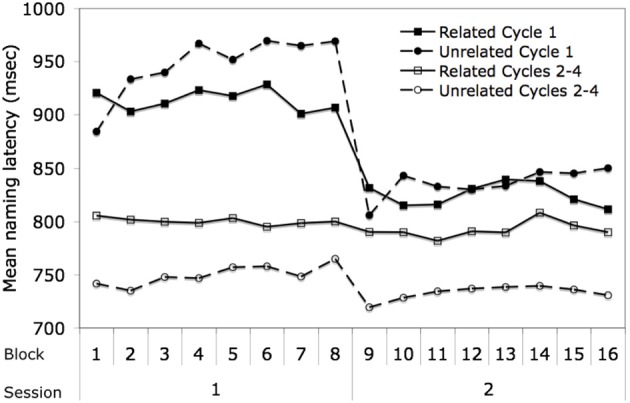
**Mean naming latency in the blocked cyclic naming task by semantic blocking condition across blocks and between sessions in the experiment**.

The results across blocks are shown in Figure 6. As indicated in the methods, within the first and second halves of the blocked cyclic naming task, eight related blocks and eight unrelated blocks were presented. Thus, the figure shows the results for the two types of blocks in terms of their order within the related and unrelated conditions, though related and unrelated blocks alternated in the actual experiment. The data are further broken down in terms of trials from cycle 1 vs. cycles 2–4, given the differing results by cycle that were obtained in the analyses by trial. As noted earlier, in the second half of the task, the items that had been presented in related blocks in the first half were presented in unrelated blocks and vice versa. An important factor to keep in mind when looking at these data is that within a task half, the items from each related block are all from different categories whereas for the unrelated blocks, there will be one item from each of eight semantic categories in each block. Thus, there is categorical overlap *across the unrelated blocks* and *within the related blocks*.

The results of ANOVAs for the factors of block, task half, and cycle 1 vs. cycles 2–4 are shown in Table 2. One obvious feature of the data is that the latencies for the first cycle decrease dramatically between the first half and the second, whereas no such trend is obvious for cycles 2–4. This observation is confirmed by highly significant cycle by task half interaction. The decrease for the first cycle across halves is not surprising, as the items in the first cycle were new items in the first half but repeated items in the second. The large facilitation from cycle 1 to cycles 2–4 in the first half was reduced considerably in the second half due to the already faster times for cycle 1 (i.e., much less was gained due to repetition in the second half given that items had already been seen in the first half). This pattern again replicates prior findings of a substantial reduction in repetition priming in subsequent repetitions after the first (e.g., Oldfield and Wingfield, 1965; Griffin and Bock, 1998).

**Table 2:**
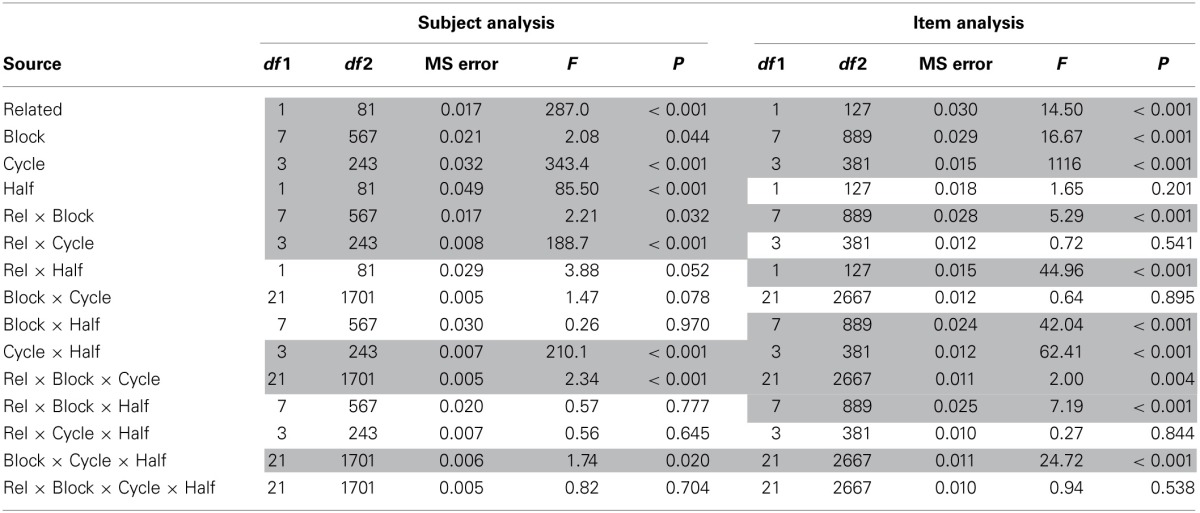
**ANOVA results for analysis of relatedness, task half, block, and cycle in the semantically blocked cyclic naming task**.

The shaded rows are those that were statistically significant at *p* ≤ 0.05.

An important feature evident in Figure 6 is that the latencies for the unrelated trials increase across blocks whereas the latencies for the related cycles stay relatively stable, which is confirmed by a significant relatedness × block interaction. The increase for the unrelated trials relative to the related trials is more evident for cycle 1 than for cycles 2–4, as reflected in a significant three-way interaction between relatedness, block, and cycle. The task half × block × cycle interaction was also significant, with latencies increasing for cycle 1 across blocks, but with much less increase across blocks in the second half. (The four way interaction of relatedness × task half × block × cycle was not close to significance, both *p*s > 0.50.) The increase in latencies across the unrelated blocks indicates a persistent effect of having named something from the same semantic category across many intervening trials, which is consistent with the learning model. The lack of effect for the related trials is also consistent with this model, since different categories of items are sampled in the different related blocks, and thus there should be no accumulating interference across blocks. The lesser increase in the unrelated condition across cycle 1 than cycles 2–4 in the first half and the lesser increase in cycle 1 in the second half as compared to the first half of the task can be attributed to the influence of repetition priming in all but the first cycle of the first half of the task.

While the increase across blocks for the unrelated condition is consistent with the learning model, the increase also highlights a problem for the model. As mentioned earlier, given the design of the blocked cyclic naming experiments (see Figure 1), items will have been presented equally often in related and unrelated conditions by the end of the experiment and latencies should thus be equivalent in both conditions (see Damian and Als, 2005). Although the times in the unrelated trials increase across blocks in the current experiment, they do not increase sufficiently such that the related and unrelated trials converge; instead, the related condition continues to have a longer mean naming latency than the unrelated condition by the end of the experiment. It should be noted that any factor related to semantic priming or better guessing of items in the related than unrelated conditions could not be the source of this remaining difference, as such should only have served to decrease rather than increase latencies in the related condition. Thus, even though it would appear that interference from learning can persist across the whole experiment, it additionally needs to be assumed that changes in connection weights decay back toward baseline over time (Damian and Als, 2005).

The increase across blocks for the unrelated items, which is largest in the first cycle, also leads to a rather unexpected explanation for the semantic priming effect observed in that cycle. The effect seems to derive from the overlap in semantic categories across unrelated blocks and the lack of such overlap across the related blocks. Thus, rather than deriving from facilitation of items within a related block, this finding suggests that the effect arises from interference which accumulates across blocks of unrelated items. In the first block of both the first half and the second half of the experiment, where such overlap across the unrelated trials cannot have yet occurred, there is no evidence of a semantic facilitation effect as latencies are longer for the related than unrelated conditions. Facilitation is not observed in the first block, where one might have most expected to see a facilitatory effect of spreading activation, as argued for by Navarrete et al. (2012).

### Summary and conclusions from blocked cyclic naming results

Our overall results replicated the often-reported effects across cycle in the blocked cyclic naming task, with either no effect or semantic facilitation in the first cycle, which turns into interference in cycles 2–4. Somewhat unexpectedly, no interactions between age and any aspect of the semantic blocking effect were obtained. In fact, even the main effect of age failed to reach significance, even when comparing the youngest of the young and the oldest of the older subjects. As mentioned earlier, Belke and Meyer (2007) had also failed to find an interaction of the blocking effect with age, and we report similar findings with a larger group of participants.

A closer analysis of the data, examining the effects of trial within cycle and effects across blocks and task halves when combining across age groups, revealed some important findings of relevance to current models of word production and the source of semantic facilitation and interference in word production. For the trials in the first cycle, there was an overall semantic priming effect, but with the effect diminishing across trials within that cycle. Potentially, this pattern could be explained in terms of a tradeoff between spreading activation at a semantic level and the changing of the strength of semantic-lexical links, with the latter having more influence as more trials are processed (Navarrete et al., 2012). However, the findings across blocks suggested a different interpretation of the facilitation effect, with the effect resulting from an increase in the unrelated times across blocks for cycle 1. This issue will be returned to after examination of the individual differences findings.

For the effects across trial within cycles 2–4, the sawtooth pattern evident in the learning model in Figure 2A, with the increase across trials within cycle as the main source of the semantic blocking effect, was clearly not in evidence. Although, there was a somewhat greater increase across trials for the semantically related than unrelated sets (1.1 ms/trial greater slope), this difference was supplemented by a large difference in the size of the repetition priming effect for related as compared to unrelated trials in the transition from cycle 1 to cycle 2. There was also a small increase across trials within cycle for the unrelated condition, which is not predicted by either the learning model or by a model incorporating learning and top-down biasing. One possible explanation for this effect is that there is some small degree of semantic overlap between items considered to be unrelated. Another possible explanation derives from the fact that, on average, items presented on earlier trials within a cycle will have appeared more recently in the preceding cycle. Thus, if one assumes some small decrease in the repetition priming effect with delay [see Brown et al. (1996), for supporting evidence from standard repetition priming experiment], the slight increase in latencies in the unrelated condition across trials could be accounted for in this way. This same factor could, of course, contribute to the increase across trials for the related trials.

## Results and discussion: correlational analysis of cognitive control measures and performance on blocked cyclic naming

The preceding analyses replicated previous semantic blocking effects and addressed the degree of fit to the incremental learning and top-down biasing accounts with some results favoring the latter, such as the decrease in the slope of naming latencies across trials beyond cycle 1. The analyses presented here directly assess the role of working memory and control processes in the semantic blocking effect taking an individual differences approach in which correlations were calculated between performance on the blocked cyclic naming task and measures of working memory (word span), response-distractor inhibition (Stroop), and the resolution of proactive interference (recent negatives). For the blocked cyclic naming task, measures of cumulative semantic interference were calculated based upon the naming latency slopes across trials averaging across cycles (trial slope), across cycles averaging across trials (cycle slope), and across blocks averaging across trials and task halves (block slope) computed separately for the related and unrelated trials. To the extent that word span is related to the ability to maintain the members of the current naming set in mind, one would expect greater spans to lead to smaller increases in interference effects. Likewise, to the extent that inhibition is related to selecting a word from semantic related competitors, one would expect the inhibition measures to be positively correlated with the degree of increase of naming latencies for the related condition across trials and cycles and for the unrelated condition across blocks. Each measure included only the data for cycles 2–4, as results from the first cycle differed considerably from those for the later trials. Reliabilities tended to be lower for the first cycle, being less than 0.21 for both relatedness conditions for trial slope for cycle 1. Reliabilities were higher for cycle slope from cycle 1 to cycle 2 (0.55 – 0.76), but this measure was of less interest in relation to other measures. For the slopes across blocks, the slopes were calculated averaging across the task halves as there were no significant differences in the slopes across task halves for cycles 2–4 for either the related [*t*_1(81)_ = 0.76, *p* = 0.45] or unrelated [*t*_1(81)_ = 1.14, *p* = 0.25] conditions.

Descriptive statistics for the two age groups on the individual differences measures are reported in Table 3. A significant difference in performance for the two age groups was observed only for the verbal Stroop task, with the younger subjects showing smaller Stroop interference. As noted earlier, these comparisons were made on log transformed data, and thus the larger effect for the older adults could not be attributed to a general slowing effect (Salthouse and Hedden, 2002). The reliabilities of all of the measures when combining across the young and old subjects were at least moderately high (i.e., greater than 0.40), except for the trial slope for the unrelated condition.

**Table 3:** **Descriptive statistics and reliabilities for measures**.

	**Younger subjects**				**Older subjects**				**Combined**	**Group difference**	
**Measure**	**Mean**	***SD***	**Range**	**Reliability**	**Mean**	***SD***	**Range**	**Reliability**	**Reliability**	***t***	***p***
**EF TASKS**											
Word span	5.4	0.8	(4,7)	–	5.3	1	(3,7)	–	–	0.92	0.36
Verbal stroop	165	61	−69,289	0.79	211	70	−64,390	0.78	0.82	3.84	<0.01
Recent negatives	147	175	(−108,783)	0.48	167	136	(−125,462)	0.36	0.44	0.84	0.40
**BLOCKED CYCLIC NAMING SLOPES (CYCLES 2–4)**											
Trial related	2.87	5.92	(−12,17)	0.61	2.68	4.73	(−5,23)	0.25	0.44	0.13	0.89
Trial unrelated	1.22	4.2	(−6,13)	0.28	0.32	4.1	(−9,12)	0.24	0.27	0.76	0.45
Cycle related	−1.5	20.25	(−52,40)	0.63	−1.1	19.12	(−39,78)	0.32	0.47	0.27	0.79
Cycle unrelated	−12	15	(−42,26)	0.55	−18	15	(−48,15)	0.49	0.53	1.53	0.13
Block related	0.5	10	(−31,20)	0.81	0.05	7.8	(−23,13)	0.64	0.74	0.23	0.82
Block related	2.6	5.8	(−12,16)	0.61	2.2	7.9	(−17,25)	0.68	0.65	0.24	0.81

### Correlations among cognitive control measures

The correlations among the cognitive control measures across both groups of subjects are shown in Table 4. For the span measure, higher numbers represented greater span and thus better performance whereas for the Stroop and recent negatives tasks, higher numbers represented greater interference and thus worse performance. None of the correlations reached significance. The lack of a correlation between Stroop and recent negatives tasks is consistent with prior claims of separable factors for resistance to distractor interference and the resolution of proactive interference (Friedman and Miyake, 2004).

**Table 4:** **Correlations among cognitive measures**.

		**Word span**	**Verbal stroop**	**Recent negatives**
Word span	*r*	−	−0.111	−0.135
	*t*	−	1.00	1.18
	*p*	−	0.318	0.23
Verbal stroop	*r*	−0.111	−	0.024
	*t*	1.00	−	0.21
	*p*	0.318	−	0.83
Recent negatives	*r*	−0.136	0.024	−
	*t*	1.18	0.21	−
	*p*	0.23	0.83	−

### Correlations between cognitive control and blocked cyclic naming measures

**Figure 7:**
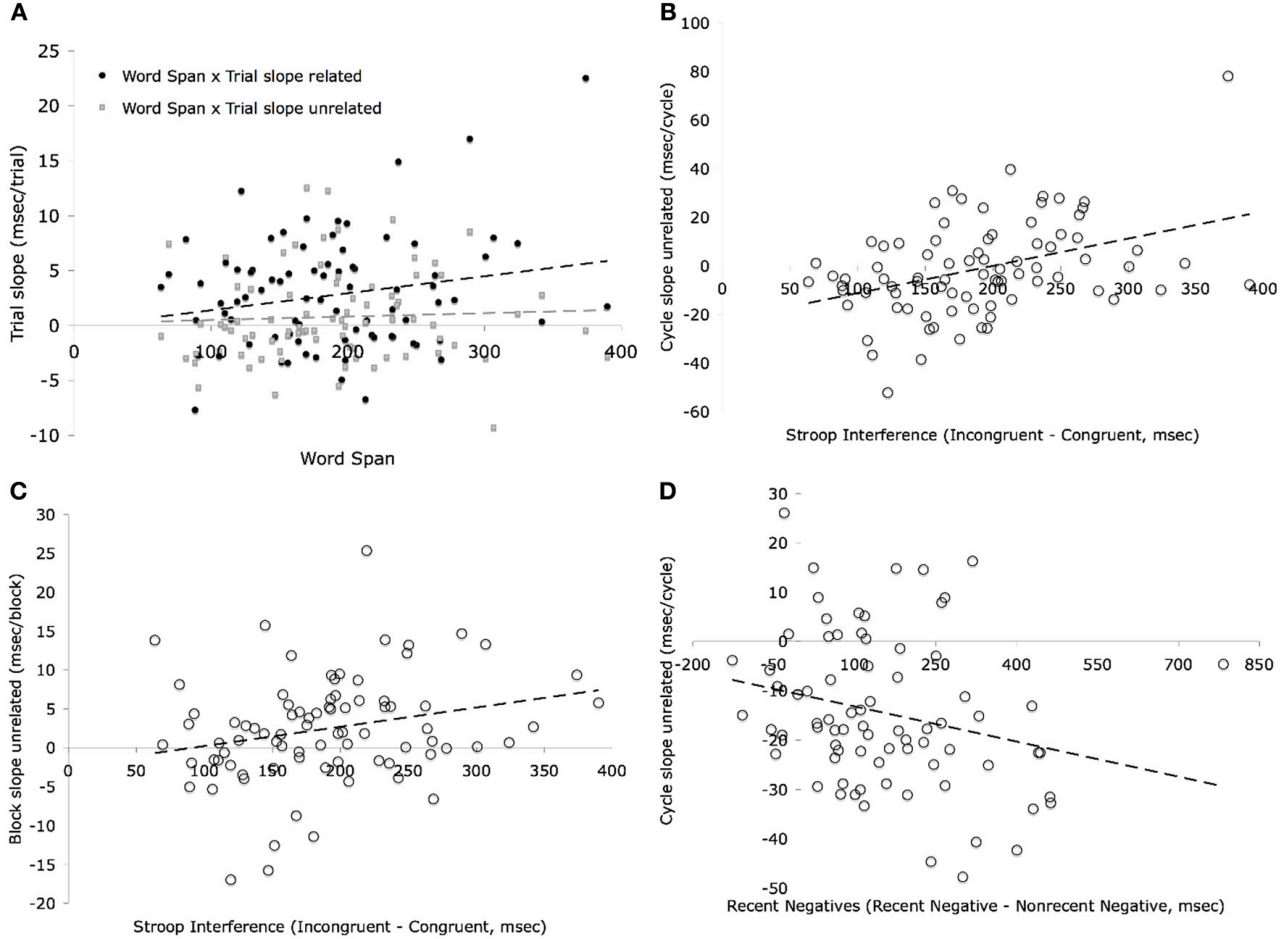
**Relationships between (A) Word span and Trial slope, (B) Stroop and Cycle slope unrelated, (C) Stroop and Block slope unrelated, and (D) Recent negatives and Cycle slope unrelated**.

The correlations between the cognitive control measures and the blocked cyclic naming measures are shown in Table 5, and scatterplots of those relationships which were significant are shown in Figure 7. The discussion below is separated into sections on correlations with trial slope within cycle, cycle slope, and block slope.

**Table 5:** **Correlations between cognitive and blocked cyclic naming slope measures for cycles 2–4**.

**Slope measure**	**Trial type**		**Word span**	**Verbal stroop**	**Recent negatives**
Trial	Related	*r*	−0.280^*^	−0.020	−0.064
		*t*	2.60	0.12	0.57
		*p*	0.011	0.905	0.560
	Unrelated	*r*	−0.218^*^	0.087	−0.114
		*t*	2.00	0.81	0.98
		*p*	0.049	0.419	0.316
Cycle	Related	*r*	−0.157	0.308^**^	−0.096
		*t*	1.42	2.88	0.83
		*p*	0.159	0.005	0.397
	Unrelated	*r*	0.101	−0.020	−0.265^*^
		*t*	0.91	0.21	2.35
		*p*	0.364	0.841	0.018
Block	Related	*r*	−0.032	0.212	0.153
		*t*	0.29	1.98	1.34
		*p*	0.774	0.051	0.170
	Unrelated	*r*	0.042	0.254^*^	0.018
		*t*	0.38	2.41	0.18
		*p*	0.706	0.018	0.852

* p < 0.05,

** p < 0.01.

#### Trial slope within cycle

To the extent that the size of the increase in latencies in the related condition relates to individuals' ability to inhibit irrelevant responses or resist interference from previous trials, one might have expected the slope across related trials within cycle to correlate positively with the Stroop measure or the recent negative measure. However, no significant correlations were obtained for either the related or the unrelated trial slopes. Instead, the slopes across trials correlated negatively with the word span measure for both the related and unrelated trials. Although the correlation was somewhat smaller for the unrelated trials than the related trials, the difference in the two correlations was not significant (*z* = 0.42, *p* = 0.67). Thus, for both the related and unrelated trials, those with a steeper negative slope across trials (i.e., increasingly rapid naming across trials) had greater working memory capacity. This finding is consistent with the top-down biasing account, which assumes that subjects maintain the current set of items (whether related or unrelated) in memory following exposure to the set in the first cycle. The negative correlation with slope suggests that subjects can anticipate what pictures would be presented towards the end of the eight trials within each cycle, thus speeding their naming latencies. Thus, the decrease in latencies derived from a process of narrowing down the set of names as the trials within a cycle progressed. This relation to word span is likely to be one, if not the major, contributing factor to the smaller slopes across trial for cycles 2–4 than cycle 1. That is, on the first cycle, subjects would not know which items were in the current set and thus response narrowing could not come into play. In fact, the correlation between span and slope across trial in cycle 1 was close to zero for both the related (*r* = −0.069) and unrelated (*r* = 0.006) conditions, but it should also be noted that the reliabilities were very low for the trial slope in cycle 1 which would make finding any relationship difficult.

#### Cycle slopes

In contrast to the results for the slopes across trials, the slopes across cycles did show some significant correlations with the Stroop and recent negatives tasks. However, a different pattern was obtained for the related and unrelated cycle slopes. Cycle slope for the related condition correlated positively with the Stroop measure, indicating that the slope of increase in naming latencies across cycles for the related condition was greater for those showing more interference on the Stroop task. The correlation for the unrelated condition was non-significant and significantly smaller than that for the related condition (*z* = 2.13, *p* = 0.03). Such a pattern is consistent with a role for inhibitory abilities in reducing the strong interference that arises in the related trials. This explanation would however imply that we should have observed a correlation of Stroop with the *trial* slope for the related condition as well. It is possible that any potential correlation with measures of inhibitory control was overridden by the effect of the narrowing of response alternatives within a cycle that was related to memory span.

For the unrelated cycle slope across cycles 2-4, there was a significant negative correlation with recent negatives. For the related cycle slope, this correlation was not close to significance [*t*_(77)_ = 0.84, *p* = 0.41], but was not different from than that for the unrelated cycle slope (*z* = 1.1, *p* = 0.27). Thus, for the unrelated condition, a greater negative slope across cycles (i.e., faster times across cycles) was associated with larger interference effects in the recent negatives task. If the recent negatives measure reflected the ability to resolve interference from related trials, a positive correlation with the related trial slope would have been expected, as was seen for the Stroop effect. The negative correlation might be interpreted as deriving from the degree of persistence of memory representations which affects the two tasks in an opposite fashion. In the recent negatives task, these persisting representations cause an increase in the familiarity of the probe item, resulting in difficulty rejecting probes matching an item from the preceding list (i.e., in the recent negatives condition) which causes an increase in the recent negatives effect. In the blocked cyclic naming task, these persisting representations for the unrelated items might aid in the naming of repeated items across blocks—that is, increase repetition priming and decrease the slope across cycles. Although the same facilitation would be expected for the related items, this repetition priming would be counteracted by the increased difficulty in selecting among related items.

#### Block slopes

For the slope across blocks, the only significant correlation was between the slope for the unrelated condition and the Stroop measure. As discussed earlier, the increase in latencies across blocks for the unrelated condition may derive from persistent changes in the semantic-lexical links resulting from prior naming of items from the same semantic category in previous blocks. This learning leads to more competition in subsequent blocks, and the resolution of this competition can be assumed to depend on similar mechanisms as involved in the Stroop task. The correlations were similar between Stroop and both the unrelated and related blocking slopes, even though the effect was only significant for the unrelated block slope. It may be the case that some smaller overlap in semantics of items across semantic categories could lead to a smaller accumulation of interference across blocks, similar to the supracategory interference effect reported by Alario and Moscoso del Prado Martín (2010). A lack of relation to the recent negatives effect can be explained on the grounds that the same items do not repeat across block for either the related or unrelated items and thus repetition priming does not play a role.

### Summary and conclusions: correlational analysis of cognitive control and blocked cyclic naming tasks

In summary, performance on the working memory, response-distractor inhibition, and proactive interference tasks related to performance on the semantically blocked cyclic naming task, though with each individual differences measure correlating with a different aspect of the task. Larger working memory capacity was related to a reduction in the cumulative increase of naming latencies across trials, affecting both related and unrelated conditions similarly (though the correlation was non-significantly smaller for the unrelated trials). Better ability to inhibit a distractor, as measured by the Stroop task, was related to lesser interference across cycles in the related condition, where a response had to be selected from highly activated competitors. Finally, a larger proactive interference effect, as measured by the recent negatives effect, was related to greater repetition priming across cycles, particularly for the unrelated condition. The contrasting pattern of correlations for Stroop and recent negatives task suggest that different mechanisms are involved in the selection of a word from strongly activated competitors (related to Stroop) and in the repetition priming effect (related to recent negatives). In the learning model of Oppenheim et al. (2010), the same parameter (learning rate) determined the degree of strengthening and weakening of semantic-to-lexical links, accounting for both repetition priming and sematic interference, whereas these results might suggest that two different parameters would be required. It is possible, though that the same parameter is involved in the weakening and strengthening of links, but the relation to Stroop derives from a separate stage of response selection. The present findings seem more consistent with the top-down biasing account (Belke and Stielow, 2013), as they proposed that separate mechanisms were involved in priming and in resolving competition.

An important question for the individual differences findings reported here is the extent to which the correlations reflect strategic processes engaged by the blocked cyclic naming task or reflect mechanisms that would be involved in word production in more naturalistic settings. We would argue that the correlations for Stroop and recent negatives reflect processes intrinsic to the naming process rather than task-specific strategies, whereas the correlations with word span would reflect task-specific, strategic processes. The version of the Stroop task employed here had very few congruent trials relative to incongruent trials. Thus, there would be little motivation for subjects to attend to the written word, as doing so would only slow down performance on the more frequent incongruent trials (Bugg et al., 2008) and thus the observed interference results from an influence of the written word despite motivation to resist its influence. In the case of the blocked cyclic naming, it seems unlikely that increasing interference across cycles in the related condition or increasing latencies across blocks for the unrelated condition comes from any strategy adopted by the subjects. For the related trials, the strategies that would seem to come to mind would be those involved in anticipating related words, which should facilitate rather than impede responses. For the unrelated trials, it seems unlikely that subjects would notice the categorical relationship between unrelated items across blocks. Thus, the correlation that comes about between Stroop and the related cyclic slope would seem to derive from an automatic influence of distractors. The correlations with the recent negatives task also seem unlikely to derive from subject strategies. In the recent negatives task, subjects have no motivation to remember the previous list items, as doing so would only impair their performance on the current list. Thus, the recent negatives effect reflects the influence of memory of a prior item despite motivation to suppress the memory. Findings in the memory literature suggest that the proactive interference effect in the recent negatives task (Öztekin and McElree, 2007) and in other proactive interference tasks (Jacoby et al., 2001) derives from a familiarity assessment rather than the recall of specific episodic information. Thus, the correlation of cycle slope with the recent negatives effect would not seem to reflect deliberate recall of prior presentations of the same item in both tasks. Instead, in both tasks, one might assume that previous presentation of an item serves to strengthen the representation of the item itself. In the recent negatives task, this strengthening increases the strength of connections between the orthographic, semantic, and phonological representations of the visually presented word [see Barde et al. (2010), for an interpretation along these lines]. In the blocked cyclic naming task, this strengthening increases the strengths of connections between the picture and its semantic representations and between the semantic representation and the lexical representation [as assumed in the Oppenheim et al. (2010) model]. As the cycle slope for the unrelated items mainly reflects repetition priming, it makes sense that this slope would relate to a measure of strengthening of representations from prior presentations.

In contrast to the correlations of cycle slope with Stroop and recent negatives, the correlations of trial slope with memory span would seem to derive from a task-specific strategy in which subjects make use of knowledge that the same items appear repeatedly across cycles and keep track of which items have already appeared within the current cycle in order to anticipate the remaining ones. The assumption of a task-specific role of working memory capacity is consistent with the findings by Belke (2008, 2013), who found that an external memory load increased the size of interference effects in blocked cyclic naming, in which items repeated, but not a continuous naming task, in which there is no fixed set of items that repeat predictably. Such a process is unlikely to be of much use in naturalistic word production situations. Although subjects in the current experiment may not have been able to remember all eight items, response times might still get faster to the extent that they could limit the response set at all as the trials within a cycle progressed. Unfortunately, the use of this strategy could serve to mask other effects, as the memory strategy would speed naming latencies as trials progressed within cycle whereas the buildup of competition would serve to increase latencies.

## General discussion

The current study employed the semantically blocked cyclic naming task to better understand the structure and functioning of the word representation system and the role of cognitive control abilities—specifically, short-term retention and inhibition—in single-word production. As discussed in the introduction, many researchers have assumed that word selection is a competitive process in which a target word must be selected from semantically related competitors and some evidence has been put forward that inhibition abilities are involved in resolving this competition (de Zubicaray et al., 2001, 2006; Guo et al., 2011; Shao et al., 2012). It should be acknowledged that some researchers have argued that selection does not occur through competition (e.g., Mahon et al., 2007) and, according to such views, a role for inhibitory abilities is not expected.

In the present study, we used the Stroop task and the recent negatives task to assess what have been purported to be separate aspects of inhibition—specifically, response-distracter inhibition and the resolution of proactive interference (Friedman and Miyake, 2004). Consistent with the separate mechanisms view, we found different patterns of correlations for the two measures. Positive correlations for the Stroop effect were observed with measures reflecting increasing competition from related items (i.e., the slope for related trials across cycle and the slope for unrelated trials across block). A negative correlation with the recent negatives task was observed for a measure reflecting repetition priming across unrelated trials.

The most common interpretation of a relation between the Stroop effect and the effects of competition is that both reflect the ability to deal with competition at the point of lexical selection. According to this approach, the mechanism involved is an attentional biasing mechanism that leads either to greater activation of the appropriate lexical representation (Schnur et al., 2006; Roelofs and Lamers, 2007) or decreased activation of related but inappropriate representations (Shao et al., 2012). Another possible interpretation of the Stroop effect (and the related picture-word interference effect, see Schnur and Martin, 2012, for discussion) is that the interference arises at a later output buffer stage where the inappropriate response (i.e., the written word name) has to be deleted so that the appropriate response (i.e., the color name) can be produced (Mahon et al., 2007). However, it is difficult to see how this interpretation could apply to the blocked cyclic task, where the representation of an inappropriate response from a previous trial (for the related cycle slope) or from a previous block (for the unrelated block slope) is unlikely to still be in a response buffer.

We have argued that the different pattern of correlations for the recent negatives effect and for Stroop resulted because the recent negatives effect could be seen as related to repetition priming—that is, in the terms of the learning model, the increase in semantic-lexical links due to prior exposure. This increase in links for a prior presentation leads to greater interference in the recent negatives task (see Barde et al., 2010) and greater repetition priming in the blocked cyclic naming task. If individual differences in the degree of strengthening of links was equivalent to individual differences in the degree of weakening of links, one would have expected to see a correlation in the opposite direction between the recent negatives task and the slope of increase across semantically related trials, but this was not observed. As discussed earlier, this might mean that there are different parameters for strengthening and weakening or, alternatively, there is one parameter for changing connection weights and another factor comes into play in selecting a response from among activated competitors. While top-down biasing provides another factor that plays a role, we would instead argue that in addition to such biasing, the ability to inhibit competitors also plays a role.

An effect of competition in word selection would be consistent both with competition deriving from persisting activation (Belke et al., 2005; Schnur et al., 2006) and competition deriving from incremental learning, involving a change in semantic-lexical links (Oppenheim et al., 2010). A number of previous studies have provided compelling support for the learning approach, given the long-lasting nature of semantic interference effects when repeatedly sampling from the same semantic category and their persistence across intervening trials (Damian and Als, 2005; Howard et al., 2006). The present study added a novel finding to this evidence—specifically, the increase in latencies for unrelated trials across blocks, as this increase resulted from the presentation of items from the same semantic category that appeared many trials earlier in a different unrelated block.

While this finding is consistent with a learning approach, there were several findings that seem to cause difficulty for the approach, at least for the particular instantiation presented by Oppenheim et al. (2010), and several of these may be accounted for better in an account that additionally assumes cognitive control in some form (i.e., Belke and Stielow, 2013). These include the following, which will be discussed in turn below:

1. A greater slope across trials within cycle for cycle 1 than for cycles 2–4.
2. A positive slope for the unrelated trials within cycle.
3. Substantially greater repetition priming from cycle 1 to cycle 2 than between subsequent cycles.
4. Much greater repetition priming from cycle 1 to cycle 2 for the unrelated than the related trials but more similar repetition priming for later cycles.
5. Non-convergence of the latencies for the related and unrelated trials at the end of the experiment.

*1. Greater slope across trials for cycle 1 than later cycles.* The predictions of the learning model (Oppenheim et al., 2010) shown in Figure 2A reveals a similar slope for the related trials across cycles and zero slope for the unrelated trials. The lesser slope for later cycles in our observed data might be accommodated on the grounds that during cycles 2–4, subjects could employ the response narrowing strategy that appeared to underlie the correlation of trial slope with memory span, whereas subjects could not employ this strategy in cycle 1. This finding does not fit with the learning account, but is a prediction made by the top-down biasing account, as subjects could use the previous knowledge of item identities to aid in overcoming interference.

*2. A positive slope for the unrelated trials within cycle.* As noted earlier, this finding could be attributed to the fact that more items would intervene between the presentation of an item in one cycle and its repetition in a subsequent cycle for items appearing later within the subsequent cycle. If so, such would suggest some diminution of repetition priming with increasing numbers of intervening items, which is consistent with some prior findings in the literature (Brown et al., 1996), but is not accommodated in the model proposed by Oppenheim et al. (2010). Assuming that repetition priming is due to the change in strength of semantic-lexical links, this finding would suggest some small degree of regression in the strength of the links over intervening trials, similar to the proposal by Damian and Als (2005).

*3. Substantially greater repetition priming from cycle 1 to cycle 2 than between subsequent cycles.* Oppenheim et al. (2010) acknowledged that although their model produced some reduction in repetition priming for unrelated trials across cycles, the decrease was substantially less than what had been observed in the blocked cyclic naming task. The present findings reinforce this difficulty for the model. Prior findings from standard repetition priming experiments on picture naming have also shown that repetition priming decreases substantially for additional repetitions after the first (e.g., Oldfield and Wingfield, 1965). The learning model does adjust the change in weights based on the prior strength of the weights, with greater changes occurring for moderately strong links than for very weak or strong leaks. However, the reduction in repetition priming would seem much more amenable to the top-down biasing account, which would predict that performance between the first cycle and subsequent ones in a block would differ because subjects would be able to utilize top-down modulation to lessen interference in later cycles but not the initial presentation cycle. That is, during the second cycle, subjects can make use of the knowledge of the items in a set gained in the first cycle to substantially decrease their name latencies. However, in subsequent cycles, little additional reduction in naming latencies can be gained from knowledge of the items in the set.

*4. Much greater repetition priming for the unrelated than for the related trials from cycle 1 to cycle 2, but more similar repetition priming for later cycles*. This finding suggests that a greater weakening of semantic-lexical links for semantically related words is needed in order to reduce repetition priming more strongly for related words relative to unrelated words. As implied by finding 3 above, subsequent adjustments need to be smaller than the first in order to account for this finding within a learning model. This finding again fits with a top-down biasing account, as the apparent repetition priming from cycle 1 to cycle 2 would instead reflect the ability of subjects to retain items in working memory and employ top-down modulation to help select the correct name on subsequent presentations. The much smaller apparent repetition priming from cycles 2–4 may in fact represent actual priming involving strengthening of connections, whereas the apparent priming from cycle 1 to cycle 2 may reflect the benefit from having the names active in working memory. It might be noted that even in the first cycle, subjects had already seen and named the pictures in the practice session, and would have seen them multiple times previously by the time they appeared in the second half of the experiment, so the differences in naming latencies between cycle 1 and 2 as opposed across cycles 2–4 could not be attributed to a larger priming effect when first encountering the item.

*5. Non-convergence of the latencies for the related and unrelated trials at the end of the experiment.* Although the model presented in Figure 2A does not show any convergence between related and unrelated trials at the end of cycle 4, such would be predicted across blocks unless the weights for the unrelated items were reset at the end of each block. Damian and Als (2005) in fact suggested that such a reset occurred, though theoretically it is unclear why such should be the case. According to a general learning model approach, there is no reason to assume that what constitutes a block in an experiment should undo learning to a pre-experimental setting. Instead, the fact that latencies remain longer for related than unrelated trials at the end of the experiment (as was the case in the present study), suggests that there is some effect of the interval between items from the same semantic category which appear consecutively in the related condition and which occur across blocks (and hence across many intervening items) in the unrelated condition. Some prior findings had suggested that such was not the case. Specifically, Howard et al. (2006) found no effect of the number of items between repeating presentations on the increase in latencies for items from the same semantic category (see also Navarrete et al., 2010). However, the persistence of the semantic blocking effect at the end of the experiment fits well with a top-down biasing account. Since this account incorporates learning, it would explain the cumulative semantic interference effects that are apparent across the course of the experiment, while also being able to account for the importance of interval between item presentations. When the items are presented in repeated sets as in the current study, top-down modulation would aid in reducing naming latencies, and this process would be more efficient for sets of unrelated items than related items as competition would still occur within the related sets. However, some findings from continuous naming studies may be problematic for this top-down biasing as a complete account of the findings. Schnur (2012) investigated the limits of cumulative semantic interference in a continuous naming paradigm. She found that semantic interference disappeared at medium (8, 10, 12, 14) and long (20, 30, 40,50) lags between semantically related items. Interference reappeared when a 2-item lag was included with the medium lags. These findings suggest that the semantic relation between items needs to be highlighted to subjects and that some attentional process is needed in order for the persisting effects to occur. Schnur's findings suggest that highlighting the semantic relationship between items to subjects leads to semantic interference, whereas the top-down biasing account predicts that such information is beneficial to subjects in overcoming interference from bottom-up learning processes.

Thus, while some of the findings from the present study suggest long-lasting learning effects, other suggest that effects may dissipate at least to some extent over intervening trials. It perhaps makes some sense that momentary changes in connection weights regress somewhat back to preceding levels (whether higher or lower) in order that momentary fluctuations in the input or the sequence of inputs do not completely override patterns learned over the long-term. A number of researchers in the learning field have suggested that learning involves some combination of fast and slow weight changes (e.g., Baddeley et al., 1988; Hauptmann and Karni, 2002; Davis et al., 2009). Such a combination of less and more permanent changes may be what underlies the patterns observed here. Fast weight changes that tend to dissipate would give more impact on latencies to a sequence of related items (within a related block) in terms of decreasing weights for items sharing semantic features than would the same sequence presented over many intervening trials (across unrelated blocks), as the fast weight changes would have dissipated in the latter case. The greater interference arising for closely presented items would provide a means of accounting for the continued interference effect for related trials observed at the end of the experiment. The notion of some decrease in weight changes over time would also provide a means of accounting for the positive slope across trials within cycle for the unrelated items. The other modification that is needed to the learning model is for greater modulation than is currently assumed of the learning effects dependent on the overall strength or weakness of existing links. This latter change would provide a means of accounting for the greater effects seen in cycle 1 and in going from cycle 1 to cycle 2 than in later cycles.

Overall, the top-down biasing account provides a better explanation of our findings without much additional modification. Because this account incorporates learning, it could explain the cumulative semantic interference effects observed both at short intervals (cycle 1 related trial slope) and over long intervals (unrelated block slope). The apparent disappearance of cumulative semantic interference in later cycles could be explained by a top-down biasing mechanism which aids in naming when the set of items to be named is known, and the semantic blocking effect itself could be explained by this biasing mechanism being less efficient when the set of items are semantically related to one another as opposed to unrelated. As noted earlier, a potential problem for this account comes from the findings reported by Schnur (2012), who found that semantic interference in a continuous naming paradigm only appeared when subjects were aware of items sharing semantic relationships. If such findings are replicated, they would be problematic for accounts based on learning and/or top-down biasing, as awareness plays no role in the learning model and leads to facilitation rather than interference in the top-down biasing approach.

Our results for the correlations with the Stroop and recent negatives measures provide further evidence that cognitive control mechanisms play a role in lexical selection, but we would argue these mechanisms do not necessarily rely upon top-down modulation by subject strategies or expectancies, instead being more automatic, and coming into play in cases of high interference. In contrast, the relationship between individual differences in working memory capacity and increasing naming latencies across trials does fit with a controlled, top-down biasing approach, as subjects would benefit from the active maintenance of information in memory for both related and unrelated items. Along the lines of the proposal by Belke and Stielow (2013) for both working memory and the biasing of relevant responses playing a role in naming, the present results support the notion of separate abilities that modulate performance in the blocked cyclic naming task independently.

## Conclusions

In conclusion, the current study addressed the source of semantic interference in naming as found in semantically blocked cyclic naming paradigms. The results failed to confirm all of the predictions of accounts of this interference as resulting from incremental changes in connection weights (Oppenheim et al., 2010). The results as a whole indicated that some regression of changes to links needs to be incorporated in order to account for all of the findings, but even with such changes, cognitive control abilities are implicated as important in the blocked cyclic naming task. Our findings favor a top-down biasing account (Belke and Stielow, 2013), which could explain both the cumulative semantic interference effects observed in the experiment as well as the modulation of such effects when subjects are able to employ more strategic processing. However, our findings with respect to the correlation of measures of semantic blocking with the inhibition tasks suggest that a more automatic inhibitory processes comes into play to help select a word from competitors under conditions of high semantic competition. This latter ability would be of more value than the expectation processes under more naturalistic production conditions.

## Author notes

This work was part of the first author's dissertation work. Portions of the research were reported at the Annual Convention of the Association for Psychological Science, May 2009. The research was supported by NIH NIDCD Grant R01DC-00218 to Rice University (PI: Randi Martin) and funding from the Rice University Psychology Department Maurin Fund. The authors would like to thank Catherine Claussen for assistance in collecting older subject data, and Camille Peres for help in allowing to test at University of Houston-Clear Lake. We would like to thank Bob Slevc and Yingying Tan for reviewing earlier versions of this document, as well as comments from two anonymous reviewers.

### Conflict of interest statement

The authors declare that the research was conducted in the absence of any commercial or financial relationships that could be construed as a potential conflict of interest.

## Footnotes

^1^The items in this figure are items used in the present study. Although the items in a related block (a row) are more visually similar than items within an unrelated block (column), prior results indicate that the semantic blocking effect persists even when the visual similarity between related items is minimized (Damian et al., 2001).

^2^Replacing outlying data points with a criterion value (termed “Winsorizing”; Barnett and Lewis, 1978) rather than eliminating them is one standard approach to the treatment of outliers (see Ratcliff, 1993, for the application to reaction time measures). Such a method is more often used in individual differences research (e.g., Miyake et al., 2000) where there may be few data points for a given condition for each subject and thus the elimination of extreme values could either distort the effect for a given subject and reduce reliabilities, or lead to the loss of the ability to use the subjects' data if all values for a given condition were eliminated. Of course, replacing extreme values could also distort the data if these extreme values did not reflect true long or short values, but instead reflected lapses of attention or inadvertent button pushes. However, if this were the case, then one would not expect replacement to improve reliability measures. In the present study, replacement vs. elimination had no effect on the significance of effects in the analyses of variance, but replacement improved reliabilities over elimination and thus led to a better ability to detect correlations of the reaction time effects with individual differences measures.

^3^A reviewer noted that, in the present study, subjects named all the pictures in the practice session, which should have reduced the change in priming between the first two cycles relative to later cycles in the experimental trials. While this seems likely to be the case, it is unclear how much such practice would reduce the effect, given the differences in procedure between naming practice and the blocked cyclic task. Of course, this issue could be addressed empirically.

## Appendix 1

Overall subject ANOVA of age, relatedness, session half, block, cycle, and trial in semantically blocked cyclic naming task

## Appendix 2

Items and categories used in the blocked cyclic naming experiment

**Table:**

**Instruments**	**Body parts**	**Clothing**	**Furniture**	**Animals**	**Vehicles**
Violin	Nose	Coat	Desk	Rabbit	Sailboat
Trumpet	Arm	Glove	Table	Pig	Train
Drum	Foot	Dress	Couch	Dog	Airplane
Guitar	Leg	Shirt	Chest	Goat	Car
Bell	Hand	Pants	Bed	Sheep	Bicycle
Harp	Ear	Hat	Stool	Horse	Truck
Piano	Eye	Belt	Chair	Mouse	Blimp
Flute	Finger	Sock	Dresser	Cat	Bus
**Appliances**	**Birds**	**Bugs**	**Foods**	**Weapon**	**Nature**
Iron	Chicken	Ant	Cake	Gun	Cloud
Radio	Duck	Bee	Cheese	Arrow	Lightning
Refrigerator	Eagle	Butterfly	Pizza	Bomb	Mountain
Telephone	Owl	Grasshopper	Sandwich	Rock	Rain
Toaster	Penguin	Fly	Bread	Cannon	Sun
Vacuum	Swan	Spider	Hamburger	Gun	Moon
Washer	Turkey	Snail	Popcorn	Rope	Tree
Television	Parrot	Worm	Spaghetti	Sword	Desert
**Tools**	**Utensils**	**People**	**Toys**		
Saw	Pot	Dentist	Ball		
Shovel	Spatula	Bride	Balloon		
Ladder	Plate	Cowboy	Doll		
Rake	Glass	King	Jumprope		
Wrench	Spoon	Priest	Kite		
Drill	Ladle	Pirate	Skateboard		
Hammer	Bowl	Waiter	Top		
Pliers	Cup	Fireman	Yoyo		
